# Rapid extrusion of perforating thumb calcinosis cutis in systemic sclerosis temporally associated with hot magnesium sulfate soaks and occlusion

**DOI:** 10.1016/j.jdcr.2026.08.036

**Published:** 2026-09-02

**Authors:** Eric L. Eisenberg

**Affiliations:** Lecturer, University of Toronto, Faculty of Medicine, Division of Dermatology

**Keywords:** calcinosis cutis, dystrophic calcification, magnesium sulfate, occlusive therapy, perforating calcinosis, systemic sclerosis

## Introduction

Calcinosis cutis is a frequent and therapeutically challenging complication of systemic sclerosis characterized by dystrophic deposition of calcium salts within damaged tissue. Distal digital lesions may perforate and extrude chalky calcium deposits, resulting in pain, ulceration, and impaired hand function.^1^^,^^2^ Management of digital calcinosis remains challenging with available therapies often only partially beneficial. Effective medical treatments remain limited and much of the available evidence derives from case reports and small series.^1^^,^^3^ This case describes imaging-confirmed perforating thumb calcinosis with rapid softening and extrusion temporally associated with hot magnesium sulfate soaks and protective occlusion.

## Case report

A 63-year-old woman with limited cutaneous systemic sclerosis (anti-RNA polymerase III positive with Raynaud’s phenomenon and sclerodactyly) diagnosed 10 years earlier presented with a lesion on the distal tip of the right thumb that developed 6 weeks before her evaluation and enlarged rapidly over the last 2 weeks (Figs 1 and 2). The lesion was minimally painful except with pressure. Her current treatment with mycophenolate mofetil remained unchanged for 2 years.

**Fig 1:**
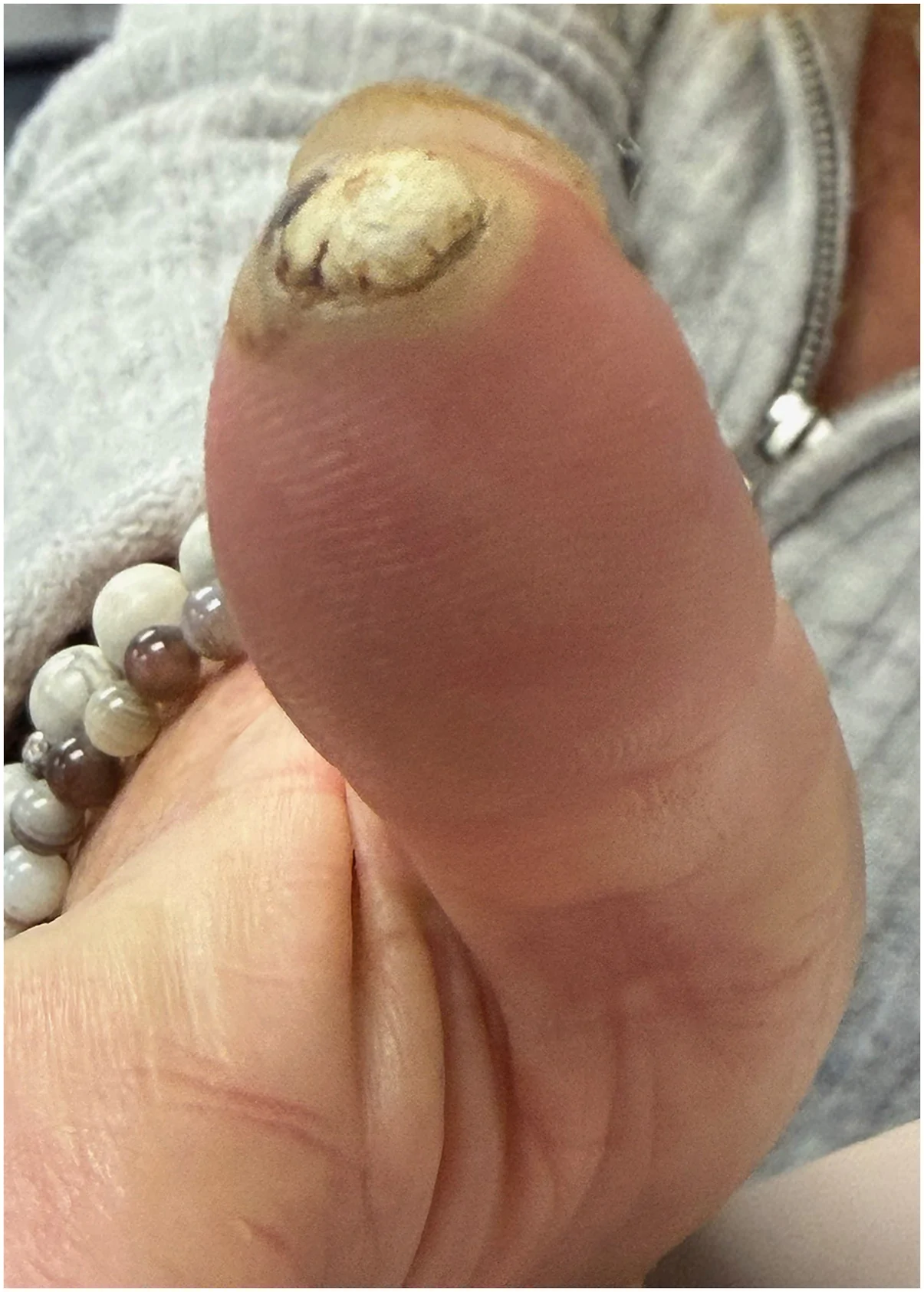
Calcinosis cutis at initial presentation - front view.

**Fig 2:**
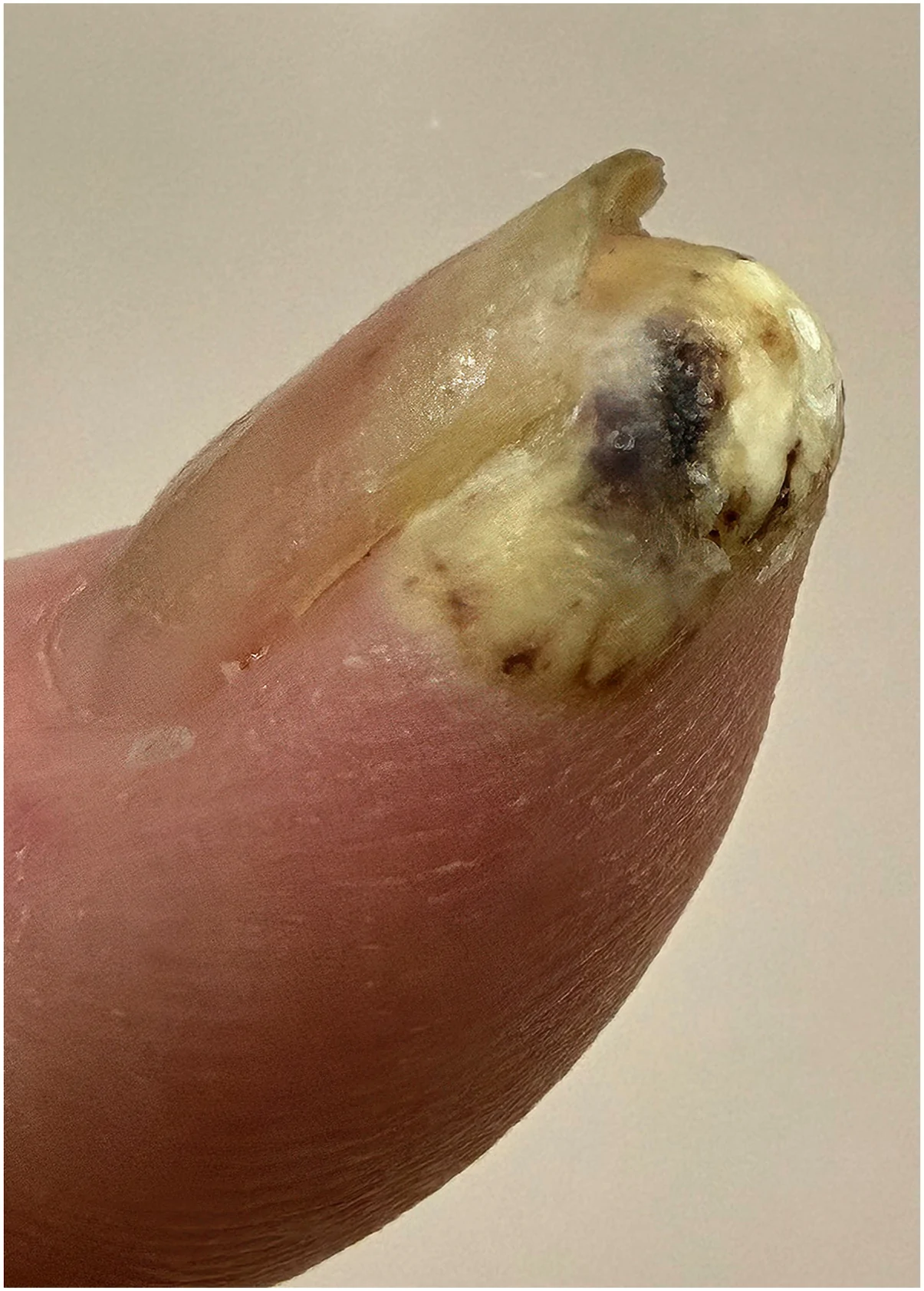
Calcinosis cutis at initial presentation - side view.

Examination showed a firm 8 mm white chalky material protruding through the thumb tip clinically consistent with perforating calcinosis cutis. She also had a 1.5 cm subcutaneous non-perforating nodule at the base of the left volar wrist.

Subsequent radiography of the thumb demonstrated extensive calcification within the volar soft tissues adjacent to the first distal phalanx, measuring approximately 2.2 cm craniocaudally. Degenerative changes were present at the first carpometacarpal, metacarpophalangeal, and interphalangeal joints. Ultrasound demonstrated multiple calcifications within the distal thumb soft tissues with mild thickening but no fluid collection or tendon involvement.

When the patient returned 5 weeks later to consider potential treatment with topical thiosulfate, she stated that based on information on a scleroderma support platform, she had begun finger soaks 1 week after her initial visit and had continued them for 4 weeks until this return visit. She used 2 teaspoons of magnesium sulfate (Epsom salts) dissolved in a bowl of hot tap water for approximately 20 minutes 1-2 times daily, and more frequently when soreness increased. She reported that the surrounding skin often became white and macerated after soaking and that more frequent soaking further reduced pain. The patient wore a rubber finger cot during daily activities to protect the affected area. She noted that after each soak the exposed calcific material progressively softened and became toothpaste-like in consistency. Small hard fragments measuring 0.5 - 3 mm gradually detached from the larger mass during the 4 weeks of soaking (Fig 3). No fragmentation had been observed prior to the soaks. A 2 mm superficial calcific spicule remained adherent to the skin. No infection, ulceration, or sensory changes were noted.

**Fig 3:**
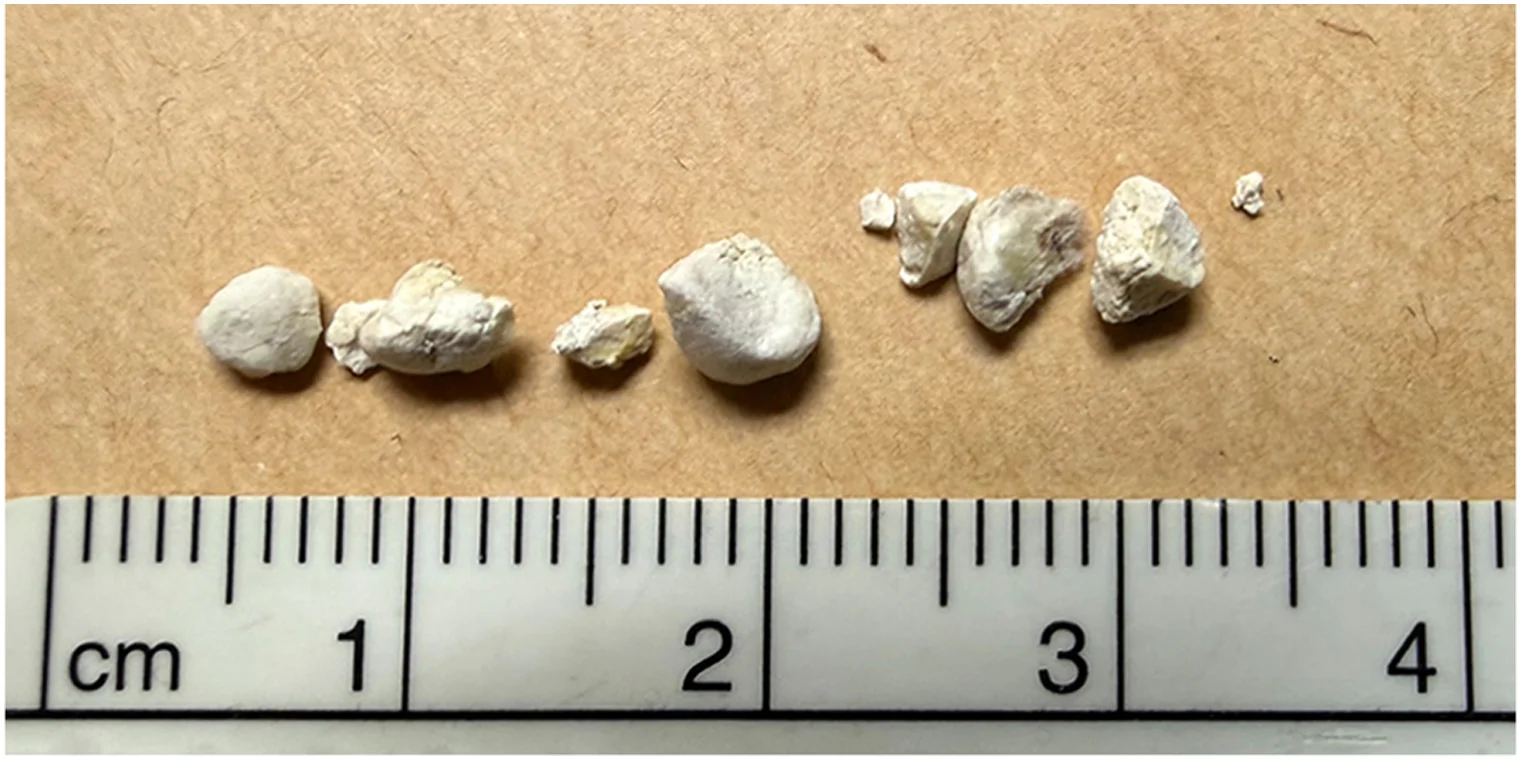
Calcinosis cutis - fragments of extruded material.

Additional imaging 10 weeks later at her second follow-up showed stability with a 3 mm reduction in craniocaudal calcification. The remaining 1 mm superficial spicule was tender to touch but was easily pared away in office (Fig 4).

**Fig 4:**
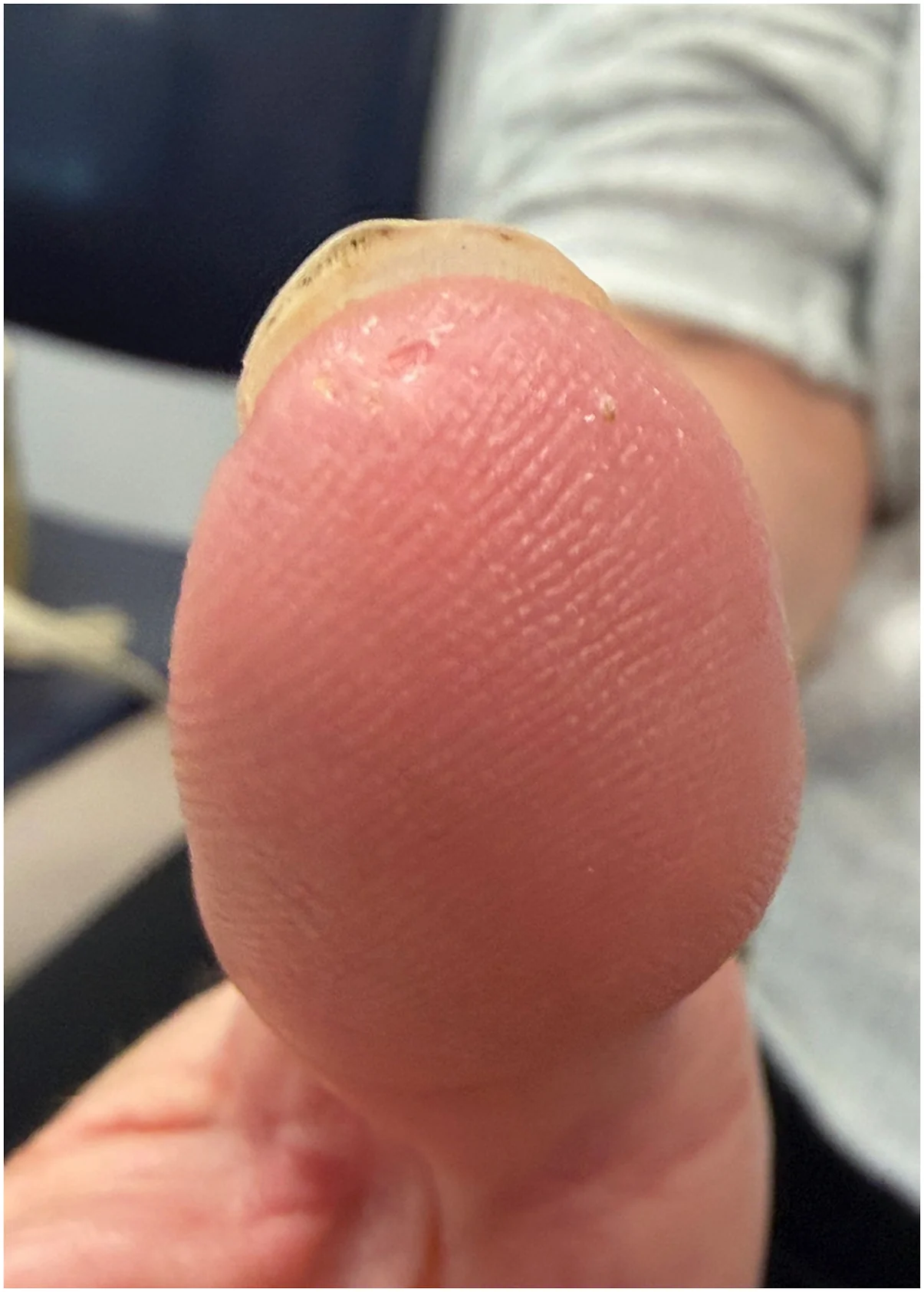
Calcinosis cutis - following complete extrusion.

## Discussion

This case demonstrates rapid softening and near-complete extrusion of perforating calcinosis temporally associated with repeated hot magnesium sulfate soaks and protective occlusion. Clinical improvement reflects resolution of the protruding component with persistence of deeper radiographic calcification.

Spontaneous extrusion is recognized in the natural history of perforating calcinosis cutis and may have contributed to the observed course.^1^^,^^2^ The magnesium sulfate soaks and subsequent occlusion may have accelerated an evolving extrusion process rather than directly altering mineral chemistry.

A variety of medical, surgical, and physical treatments has been reported for calcinosis cutis, although evidence remains limited and largely derives from small studies and case reports.^3^ Sodium thiosulfate is the most frequently reported treatment and is thought to increase calcium solubility through formation of soluble calcium thiosulfate complexes.^3^, ^4^, ^5^ More recent in vitro work has also explored alternative chelators such as citric acid.^6^ In contrast, magnesium salts are supported experimentally as inhibitors of hydroxyapatite nucleation and crystal growth rather than as agents capable of dissolving established calcific deposits.^7^^,^^8^ The observed course therefore may reflect hydration-facilitated mechanical extrusion although spontaneous extrusion cannot be excluded since the lesion was already perforating at presentation. Accordingly, this observation should be regarded as hypothesis generating rather than evidence of a causal therapeutic effect.

The striking contrast between extensive radiographic calcification and rapid clinical resolution of the perforating component underscores the dynamic and compartmentalized behavior of calcinosis cutis, in which superficial deposits may evolve independently of deeper calcified tissue.

The exposed calcific material was attached only at the peripheral epidermal margins and by deeper anchoring within the soft tissue. Hot hydration likely softened the surrounding keratinized tissue at these attachment points, facilitating progressive loosening and detachment of the superficial calcified material. The patient's observation that the exposed calcification became softer and toothpaste-like with soaking suggests hydration also altered its physical consistency. The protective finger cot likely prolonged moisture exposure, enhancing hydration and epidermal maceration while reducing mechanical trauma.^9^ Together, these effects may have destabilized both the anchoring interface and the superficial perforating calcified mass, promoting progressive fragmentation and mechanical extrusion of calcific debris.

The patient’s reporting of reduced soreness during and after soaking and greater relief with more frequent soaking may have reflected local soothing effects of hot water exposure and, possibly, anti-inflammatory effects of magnesium-rich bathing, although evidence supporting this mechanism in calcinosis cutis is indirect and limited.^10^

For safety, patients attempting this approach should use comfortably warm rather than hot water, begin with 5 minutes and gradually increase to 20 minutes 1-2 times daily, inspect the skin regularly for thermal injury, and undergo physician follow-up to detect sensory changes, infection, or impaired healing.

Systemic sclerosis-associated calcinosis arises from chronic vascular dysfunction, tissue hypoxia, and impaired wound remodeling.^1^^,^^3^ Conservative measures that reduce local trauma and maintain hydration may facilitate extrusion of superficial perforating lesions and reduce the need for invasive procedures. Further study is warranted to determine whether hydration-based approaches can safely promote fragmentation and extrusion of superficial perforating calcinosis in select patients.

### Declaration of generative AI and AI-assisted technologies in the writing process

During the preparation of this work the author used ChatGPT to ensure conformity with the formatting style of the JAAD and to provide potential references related to this disease. After using this service, the author reviewed and edited the content as needed and takes full responsibility for the content of the published article.

## Conflicts of interest

None disclosed.
